# Further evaluation of plasma sphingomyelin levels as a risk factor for coronary artery disease

**DOI:** 10.1186/1743-7075-3-5

**Published:** 2006-01-05

**Authors:** Axel Schlitt, Stefan Blankenberg, Daoguang Yan, Hans von Gizycki, Michael Buerke, Karl Werdan, Christoph Bickel, Karl J Lackner, Juergen Meyer, Hans J Rupprecht, Xian-Cheng Jiang

**Affiliations:** 1 Department of Anatomy and Cell Biology and Scientific Computing Center, State University of New York, Downstate Medical Center, Brooklyn, USA; 2 Department of Medicine II and Institute of Clinical Chemistry and Laboratory Medicine, Johannes Gutenberg-University Mainz, Germany; 3 Department of Medicine III, Martin Luther-University, Halle-Wittenberg, Germany

## Abstract

**Background:**

Sphingomyelin (SM) is the major phospholipid in cell membranes and in lipoproteins. In human plasma, SM is mainly found in atherogenic lipoproteins; thus, high levels of SM may promote atherogenesis.

**Methods:**

We investigated in a median follow up of 6.0 years the association of SM with the incidence of a combined endpoint (myocardial infarction and cardiovascular death) in stable and unstable patients, and its relation to other marker of atherosclerosis in 1,102 patients with angiographically documented CAD and 444 healthy controls.

**Results and discussion:**

Logistic regression analysis showed that SM categorized by median was associated with an elevated risk for CAD (HR 3.2, 95%CI 2.5–4.0, p < 0.05). SM levels were correlated with apoB (r = 0.34) and triglyceride levels (r = 0.31). In patients with stable angina (n = 614), SM categorized by median was not related to incidence of a combined endpoint (cardiovascular death and myocardial infarction) (p = 0.844 by Log-rank test). However, in patients with acute coronary syndrome (n = 488), elevated SM was related to the combined endpoint (p < 0.05 by Log-rank test), also in a multivariate Cox regression analysis including potential confounders (HR 1.8, 95%CI 1.0–3.3, p < 0.05).

**Conclusion:**

The results of our study reveal that 1) human plasma SM levels are a risk factor for CAD; 2) the pro-atherogenic property of plasma SM might be related to metabolism of apoB-containing or triglyceride-rich lipoproteins; and 3) plasma SM levels are a predictor for outcome of patients with acute coronary syndrome.

## Introduction

Atherogenesis is initiated by the interaction of cholesterol-rich lipoproteins with the arterial wall [1]. Many processes have been implicated in early atherogenesis, including lipoprotein oxidation [2], lipoprotein retention and aggregation [1,3], endothelial alteration, macrophage chemotaxis and foam cell formation, and smooth muscle cell migration and alteration [4]. However, subendothelial retention and aggregation of atherogenic lipoproteins have emerged as the primary pathogenic processes [1].

Two sets of evidence indicate that aortic and plasma sphingomyelin (SM) levels are closely related to the development of atherosclerosis: First, it has long been known that SM accumulates in atheromas formed in humans and in animal models [5-7]. The low-density lipoprotein (LDL) extracted from human atherosclerotic lesions is much richer in SM than the LDL from plasma [8,9]. A substantial amount of the SM found in arteries and atherosclerotic lesions appears to arise from synthesis in arterial tissue [10]. The SM concentration is also significantly increased in macrophages treated with acetyl-LDL plus an acyl-CoA:cholesterol acyltransferase inhibitor [11]. However, even in atherosclerotic lesions, the apparent rate of SM formation is relatively low compared with the rate of total phospholipid synthesis [12], suggesting that other factors might also contribute to intimal SM accumulation. Secondly, plasma SM levels are increased in human familial hyperlipidemias, especially in familial hypercholesterolemia [13], and also in animal models of atherosclerosis [14-16]. The concentration of SM is an important determinant of the susceptibility of lipoprotein SM to sphingomyelinase (SMase) [9,16], which might in turn cause atherogenic lipoprotein aggregation and atherosclerosis [17]. These findings suggest that plasma SM levels may be a risk factor for atherosclerosis.

In order to further investigate the role of SM in atherosclerosis, we measured plasma SM levels in 1,102 patients with angiographically proven coronary artery disease (CAD) and 444 healthy controls and its relationship to other risk factors and clinical outcome.

## Methods

### Study population

Between November 1996 and July 2000, we recruited 1,102 patients suffering from symptoms of CAD (614 patients with stable angina [SAP]; 488 with acute coronary syndrome [ACS]) admitted to the second medical department of the Johannes Gutenberg-University Mainz or the Bundeswehrzentralkrankenhaus Koblenz for diagnostic coronary angiography. The sole inclusion criterion was the presence of a stenosis > 30% in at least one major coronary artery. The study is described in detail elsewhere [18]. Exclusion criteria were lack of CAD as defined above and evidence of significant concomitant disease, in particular severe valvular heart disease, known cardiomyopathy, malignancy, inflammatory diseases, or a febrile condition. Patients completed a questionnaire about smoking habits, history of diabetes mellitus, hypertension, hyperlipoproteinemia, current drug use, and family history of premature CAD (documented in one first-degree relative before age 65). Diabetes mellitus was diagnosed in patients who had previously undergone dietary treatment or received additional oral antidiabetic or insulin medication or who had a current fasting blood sugar level > 125 mg/dl; hypertension was diagnosed in patients who had received antihypertensive treatment or had been diagnosed as hypertensive (blood pressure > 160/90 mmHg); hyperlipoproteinemia was diagnosed in patients who had been given lipid-lowering medication or had a history of cholesterol levels > 240 mg/dl.

Patients were followed up for a median 6.0 years. The majority of the patients presented at our clinic for follow-up; a small number of them were interviewed by telephone by trained medical staff. At follow-up, information was obtained about cardiovascular death or nonfatal myocardial infarction (n = 163). Information about the cause of death or clinical events was obtained from hospital or general practitioner charts.

Healthy control subjects (n = 444) were recruited either from general practitioners' offices in the course of routine check-up visits or by newspaper announcement, which briefly described the study design and invited healthy individuals aged ≥40 years to participate in this study as control subjects. Of the individuals who presented, we selected those without any clinical or anamnestic evidence of atherosclerosis and without evidence of any pathological ECG pattern. All individuals who presented received reports of any classical and treatable risk factors for personal use.

In general, study and control patients were of German nationality and were inhabitants of the Rhein-Main Area. The study was approved by the ethics committee of the University of Mainz. Participation was voluntary, and each study subject gave written informed consent.

### Laboratory methods

Blood was drawn from all subjects under standardized conditions after an overnight fasting period and before coronary angiography was performed. Samples were placed on ice immediately and within 30 minutes were centrifuged at 4,000 rpm for 10 minutes, divided into aliquots, and frozen at -80°C until analysis.

Plasma SM levels were measured as described previously [19]. SM was analyzed concurrently in patients and controls and the laboratory personnel was unaware of the individual's study allocation.

Plasma lipid levels (total cholesterol, Roche Diagnostics, Germany; high-density lipoprotein cholesterol (HDL-C), Rolf Greiner Biochemica, Flacht, Germany; LDL-C, calculated according to the Friedewald formula; triglycerides, Roche Diagnostics, Germany) were determined immediately. Apolipoprotein A-I (apoA-I) and apoB concentrations were determined by immunoturbodimetric assays (Tina-quant, Roche Diagnostics, Germany). The lipoprotein(a) [Lp(a)] concentration was determined using an enzyme-linked immunosorbent assay-based method supplied by Immuno Ltd. (Dunton Green, Kent, UK). C-reactive protein (CRP) was determined by a highly sensitive, latex particle-enhanced immunoassay (detection range of 0 to 20 mg/l); the between-day imprecision of this assay (n = 21) was 2.14% and 1.44% at mean levels of 1.90 mg/l and 4.33 mg/l (Roche Diagnostics, Germany).

### Statistical analysis

Demographic and clinical variables of cases and controls were compared by Chi square test for categorical and ANOVA test for continuous variables. Because of skewed distribution for triglyceride and Hs-CRP, median values were presented and the Mann-Whitney test applied for these variables. The distributions for plasma concentrations of SM were skewed and all analyses were calculated with log-transformed values; however, exponential values are presented. For correlation of SM with further variables, Pearson's correlation coefficients were calculated, using log-transformed values of SM, triglycerides and high-sensitive CRP (Hs-CRP) for each group individually. Case and control r-values were compared using Fisher's Z-transformation. We aimed to assess any evidence of association between SM and CAD in models, assuming both linear and nonlinear effects. We thus divided cases and controls into two groups stratified by median (48.1 mg/dl) on the basis of the overall study population and applied a logistic regression model.

Survival was analyzed by the Kaplan-Meyer method and Log-rank test. In all survival analyses, the endpoint was a combined endpoint of death of cardiovascular causes and nonfatal myocardial infarction. Data on patients who died of other causes were censored at the time of death. The association of SM with outcome was evaluated by Cox regression analyses adjusted for potential confounders. In these analyses, SM was considered in two groups stratified by median; other markers were considered either as continuous (log-transformed) or categorical variables. Hazard ratios (HRs) and 95 % confidence intervals (CIs) are reported with 2-tailed probability values. P < 0.05 was considered to be significant. All analyses were carried out using SPSS 11.5 software.

## Results

### Demographic and clinical measures

Baseline data regarding case and control subjects are outlined in Table 1. As expected, the prevalence of classical risk factors such as current smoking (27.3% versus 11.0%), history of diabetes mellitus (21.6% versus 3.7%), hypertension (71.9% versus 28.6%), and family history of premature CAD (38.2% versus 20.1%) was more frequent in cases than in controls. Furthermore, a significantly higher rate of ACE inhibitor (47.8% versus 8.3%) and statin (35.8% versus 6.3 %) prescriptions was noted in cases than in controls. The higher prescription of statins might well have caused the significantly lower levels of total cholesterol and LDL-C levels noted in the cases. HDL-C and apoA-I levels were lower in cases than in controls, as expected. Lp(a) and triglycerides but not apoB levels were higher in the patients. Phospholipid transfer protein (PLTP) and cholesterol ester transfer protein (CETP) activity and Hs-CRP concentrations were also elevated in patients.

**Table 1:** Demographic and clinical measures in cases and controls. For cases and controls categorical variables are presented as percentage of patients; chi-square test was used for group differences; and continuous variables are presented as mean values (standard deviation) or *median values (25/75th interquartiles) because of skewed distribution. The p values were obtained by *Mann-Whitney test. #: data for 913 patients for ejection fraction, data for 200 controls/1026 patients for apoA-I and apoB, for 200 controls/905 patients for Hs-CRP, and for 200 controls/1060 patients for Lp(a).

**Variable**	**Cases n = 1102**	**Controls n = 444**	**P- value**
**Age, years**	61.1 (10.1)	59.7 (7.4)	<0.001
**BMI, kg/m2**	27.2 (3.7)	26.7 (4.3)	0.02
**Sex, male %**	74.7	72.7	0.43
**Current smoking, %**	27.3	11.0	< 0.001
**Diabetes mellitus, %**	21.6	3.7	<0.001
**Hypertension, %**	71.9	28.6	<0.001
**Family history of CAD, %**	38.2	20.1	<0.001
**Statins, %**	35.8	6.3	< 0.001
**ACE inhibitors, %**	47.8	8.3	<0.001
**Angiotensin receptor blockers, %**	5.0	3.8	0.327
**Ejection fraction <40%#, %**	7.6	-	-
Hs-CRP*, mg/l#	4.4 (1.9/12.4)	1.5 (0.9/3.8)	<0.001
**PLTP, U/l**	25.5 (9.8)	22.4 (7.4)	<0.001
**CETP, U/l**	49.9 (20.9)	41.4 (17.4)	<0.001
**Total cholesterol, mg/dl**	219.7 (45.7)	238.4 (41.8)	<0.001
**LDL cholesterol, mg/dl**	141.0 (40.1)	155.5 (35.1)	<0.001
**HDL cholesterol, mg/dl**	48.8 (14.7)	59.4 (15.9)	<0.001
Triglyceride*, mg/dl	141 (102/193)	120 (87/167)	<0.001
**Apolipoprotein A-I, g/l#**	1.31 (0.24)	1.62 (0.28)	<0.001
**Apolipoprotein B, g/l#**	1.20 (0.28)	1.19 (0.23)	0.3
**Lipoprotein(a), mg/dl#**	36.3 (38.9)	24.2 (30.4)	<0.001

### Relationship between SM and CAD

**Figure 1:**
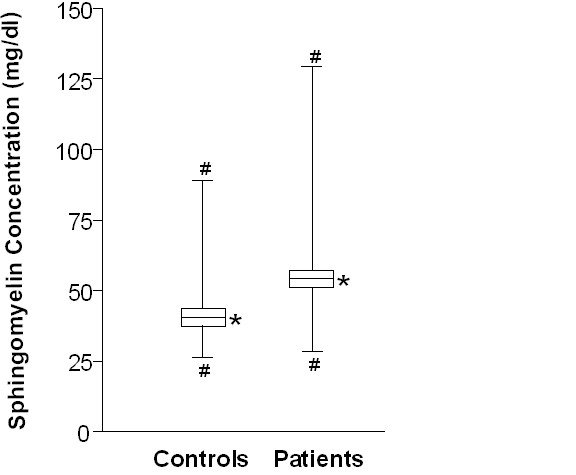
Plasma SM concentrations in patients and controls (p < 0.001 by ANOVA). *: mean with 95% confidence intervals, #: minimum/maximum.

Figure 1 shows that patients with CAD had higher plasma SM levels than healthy controls (mean 51.8 mg/dl versus mean 44.9 mg/dl; p < 0.001 by ANOVA).

Logistic regression analysis showed that plasma SM categorized by median was associated with an elevated risk for CAD (HR 3.2, 95%CI 2.5–4.0, for subjects with SM ≥48.1 mg/dl compared to subjects with SM < 48.1 mg/dl).

### Correlation with other risk factors

To investigate the possibility that plasma SM levels could act as a marker for triglyceride-rich lipoproteins or apoB-containing lipoprotein metabolism, we performed correlation analyses as outlined in Table 2. In all subjects correlations were found between plasma SM and apoB (r = 0.34) and between plasma SM and triglycerides (r = 0.31). Moreover, these relationships were significantly stronger in patients than in controls (for apoB, r = 0.36 for patients and r = 0.25 for controls, respectively, p = 0.03; for triglyceride, r = 0.32 for patients and r = 0.11 for controls, respectively, p < 0.001, see Table 2).

**Table 2:** Pearson coefficients of correlation between SM and other continuous variables (age, further lipid variables, CETP, PLTP, and Hs-CRP), stratified by CAD. Comparison of r-values of cases and controls by Fisher's Z-transformation (p- and Z-values presented)

**Sphingomyelin**‡				
	**All**	**Cases**	**Controls**	**p-value (Z-value)**

**Age, years**	- 0.03	0.06	-0.03	0.11 (1.6)
**BMI, kg/m^2^**	0.08	0.09	0.06	0.59 (-0.54)
**Total cholesterol, mg/dl**	0.17	0.25	0.14	0.04 (2.03)
**LDL cholesterol, mg/dl**	0.13	0.20	0.13	0.2 (1.28)
**HDL cholesterol, mg/dl**	- 0.14	-0.08	-0.01	0.12 (-0.24)
**Triglyceride, mg/dl‡**	0.31	0.32	0.11	<0.001 (3.92)
**Apolipoprotein A-I, g/l**	-0.05	0.05	-0.07	0.03 (2.13)
**Apolipoprotein B, g/l**	0.34	0.36	0.25	0.03 (2.15)
**Lipoprotein(a), mg/dl**	0.03	0.01	-0.04	0.37 (0.89)
**CETP, U/l**	-0.11	-0.03	-0.19	< 0.001 (2.88)
**PLTP, U/l**	0.01	-0.06	0.08	0.01 (-2.94)
**Hs-CRP, mg/l‡**	0.05	0.05	0.12	0.21 (1.25)

‡: log-transformed variables

Weak associations were found between plasma SM levels and total cholesterol (r = 0.17), LDL-C (r = 0.13), HDL-C (r = -0.14) and CETP (r = -0.11) in all subjects. The correlations between SM and total cholesterol, LDL-C, and HDL-C were stronger in patients; however, the correlation between SM and CETP was stronger in controls.

There were no or only very weak associations between plasma SM levels and other risk factors, including age, BMI, apoA-I, PLTP, and Lp(a). It is of interest that SM concentrations were not related to Hs-CRP levels (r = 0.05).

For the possible relationship of plasma SM to categorical risk factors, ANOVA tests were performed separately for patients and controls. Statin intake seemed to influence plasma concentrations in patients, but a significant trend for lower plasma SM concentrations was not observed in patients receiving statin treatment (p = 0.068). Sex, diabetes (in patients), and left ventricular ejection fraction (dichotomized for <40%) did not affect the concentration of plasma SM (see Table 3). The small number of controls with diabetes might explain the significantly elevated SM plasma concentrations in this subgroup. We found a trend for higher plasma SM concentrations in smokers. However, SM was significantly decreased in patients receiving ACE inhibitors (51.3 mg/dl versus 49.7 mg/dl, p = 0.015) but not in controls taking this medication (44.3 mg/dl versus 42.8 mg/dl, p = 0.309) (Table 3).

**Table 3:** Mean of plasma SM levels according to cardiovascular risk factors.

	**Controls**			**Patients**		
	n	SM in mg/dl	p-value	n	SM in mg/dl	p-value

**Sex**						
female	121	44.7 ± 1.2	0.398	279	51.3 ± 1.25	0.210
male	323	43.9 ± 1.21		823	50.3 ± 1.24	
**Diabetes mell.**						
-	428	43.9 ± 1.21	<0.001	863	50.7 ± 1.24	0.362
+	16	52.8 ± 1.26		239	49.9 ± 1.26	
**Hypertension**						
-	317	44.0 ± 2.29	0.628	309	51.5 ± 1.25	0.077
+	127	44.4 ± 1.24		793	50.2 ± 1.25	
**Smoking**						
-	395	43.9 ± 1.21	0.048	801	50.2 ± 1.24	0.057
+	49	46.4 ± 1.21		301	51.6 ± 1.26	
**Statins**						
-	411	44.0 ± 1.21	0.46	690	51.0 ± 1.24	0.068
+	28	45.3 ± 1.19		395	49.7 ± 1.26	
**ACE inhibitors**						
-	407	44.3 ± 1.21	0.309	575	51.3 ± 1.26	0.015
+	37	42.8 ± 1.26		527	49.7 ± 1.23	
**Ejection fraction**						
<40%	-	-	-	69	50.4 ± 1.24	0.325
>40%	-	-		844	51.4 ± 1.21	

### Cardiovascular risk in patients

In patients with stable angina, SM was not related to the combined endpoint (p = 0.844 by log-rank test, see Figure 1a). However, in a group of patients with acute coronary syndrome and plasma SM concentrations above the median (48.1 mg/dl), a higher proportion of patients with myocardial infarction and cardiovascular death was found (p < 0.05 by Log-rank test, see Figure 1b). For further statistical evaluation of these data, univariate (model 1, Table 4) and multivariate (model 2, model 3, Table 4) Cox regression analyses were performed. The multivariate model including age, sex, body -mass index, smoking, diabetes mellitus, arterial hypertension, hyperlipoproteinemia, family history of CHD, statins, log-transformed triglycerides, and apoB showed that patients with ACS and SM ≥48.1 mg/dl were at an elevated risk for further cardiovascular events (HR 1.8, 95%CI 1.0–3.3, p = 0.048, Table 4).

**Table 4:** Cox regression analyses of SM stratified by median (48.1 mg/dl) in patients with acute coronary syndrome in univariate (model 1) and multivariate models (model 2 included age, sex, BMI, smoking, diabetes mellitus, arterial hypertension, and family history of CHD; model 3 further adjusted for statins, apoB, and log-transformed triglyceride)

	**SM <48.1 mg/dl**	**SM ≥48.1 mg/dl**	**P-value**
**OR Model 1**	---	0.6	0.049
**95%CI**		0.3–0.9	
**OR Model 2**	---	1.9	0.019
**95%CI**		1.1–3.4	

**OR Model 3**	---	1.8	0.048
**95%CI**		1.0–3.3	

## Discussion

In the present study we confirmed our previous observation that plasma SM levels are a risk factor for CAD [19]. Furthermore, we revealed that plasma SM levels showed the strongest correlation with plasma apoB and triglycerides levels, suggesting that human plasma SM levels could be a marker for triglyceride-rich or apoB-containing lipoprotein remnant accumulation found in human atherosclerosis as we suggested previously [20]. Moreover, we found that plasma SM levels are a predictor for event-free survival of patients with acute coronary syndrome, suggesting a possible link between plasma SM levels and the instability of coronary plaques. We also found a not significant trend for lower plasma SM concentrations in patients under statin treatment. Diabetes mellitus or low ejection fraction did not affect plasma SM concentrations.

Why is SM related to the atherosclerotic process? SM might be a marker for an inflammatory effect, and inflammatory markers such as CRP have been shown to be important risk factors for atherosclerosis [21]. However, plasma SM levels did not correlate with Hs-CRP in our study (Table 2). Thus, it is unlikely that SM acts as a surrogate inflammatory marker.

Plasma SM levels are related to atherogenic lipoprotein aggregation, which is the initial step in the development of atherosclerosis [17]. It is well known that SM content is much higher in apoB-containing or triglyceride-rich lipoproteins than in HDL [22]. This may indicate that apoB-containing or triglyceride-rich lipoproteins are atherogenic but that HDL is not. It is also well known that aggregated lipoproteins are prominent in atherosclerotic lesions [3,23-26]. In addition, processes that promote lipoprotein aggregation before or during retention dramatically increase the amount of lipoprotein retained [27,28], which, in turn, might amplify the atherogenic responses to this retention [1,28]. The mechanisms responsible for increased retention as a result of aggregation include increased binding of the aggregates to the subendothelial matrix [27,28] and decreased efflux of the aggregates from the arterial wall due to their increased size [30,31]. Atherogenic lipoprotein aggregation after hydrolysis by sphingomyelinase is the most physiologically plausible mechanism [32,9,34]. Hydrolysis of approx. 25% of the SM of LDL to ceramide in vitro by bacterial SMase leads to the formation of aggregated and fused particles that are excellent inducers of macrophage foam cell formation [28,32,34]. There is evidence to show that a mammalian arterial-wall SMase may carry out a similar reaction in atherosclerotic lesions in vivo [33]. Schissel et al. [35,36] discovered a novel form of SMase called secretory SMase, or S-SMase. Although S-Smase is secreted by macrophages, it is secreted even more abundantly by endothelial cells [37]. Thus, S-SMase could be present in arteries before lesions develop and therefore might explain very early lipoprotein aggregation [3]. Further evidence implicating a role for S-SMase in lipoprotein aggregation in atherosclerotic lesions includes its ability to hydrolyze and aggregate atherogenic lipoprotein [9], its increased secretion from endothelial cells treated with cytokines that have been implicated in atherogenesis [39], and its presence in atherosclerotic lesions as demosntrated by immunohistochemistry [38].

As we suggested previously [20], SM might be a surrogate marker for the metabolism of triglyceride-rich and apoB-containing lipoproteins. In the present study, we found the strongest correlations between plasma SM and apoB or triglycerides (Table 2). Humans spend most of their lives in the postprandial state. The long-term effect of repeated, prolonged, and exaggerated bouts of alimentary lipemia may be disturbances in the metabolism of endogenous lipoproteins that are rich in SM and atherogenic [39]. It is conceivable that postprandial SM remnants in apoB-containing or triglyceride-rich lipoproteins may not only serve as a clearance marker of these particles, but also have their own impact on atherosclerosis. If somehow the clearance of postprandial apoB-containing- or triglyceride-rich lipoproteins was blocked, the SM-rich particles could likely be aggregated in the arterial wall after encountering sphingomylinase there, and development or instability of atheroscerotic plaques could be the consequence. However, epidemiological studies of postprandial SM levels and atherosclerosis are still lacking.

Moreover, atherosclerotic LDLs exhibit an increase in SM [9], which is believed to stimulate hydrolysis of LDL by secreted Smase. In addition, the ceramide content of lipoproteins found in atherosclerotic lesions is increased (up to a 50-fold enrichment as compared with plasma LDL) [36]. These two mechanisms may be further involved in apoptosis of affected cells such as smooth muscle cells or macrophages via the SM/ceramide pathway, resulting in destabilization of atherosclerotic plaques.

## Conclusion

Overall, plasma SM is risk factor for CAD and a risk factor for further cardiovascular events in patients with acute coronary syndrome. Its relationship to apoB-containing or triglycerides-rich lipoproteins as shown in our data could partly explain its proatherogenic properties.

## Competing interests

The author(s) declare that they have no competing interests.

## Authors' contributions

All authors have made substantial contributions to conception and design, or acquisition of data, or analysis and interpretation of data. All authors have been involved in drafting the manuscript or revising it critically for important intellectual content, and have given final approval of the version to be published.

**Figure 2:**
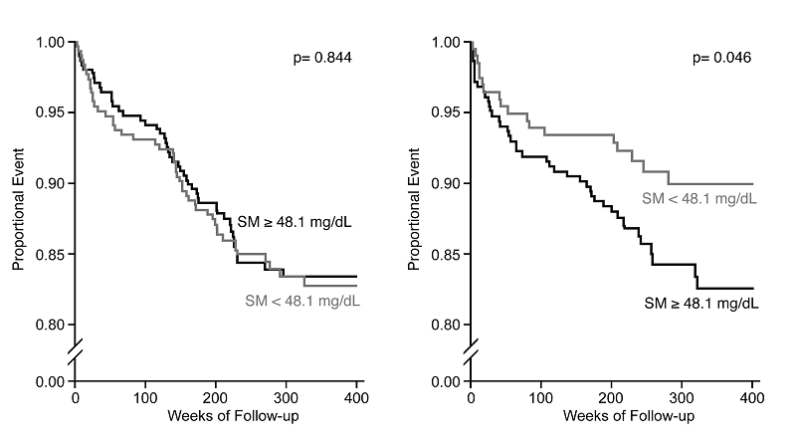
Kaplan-Meyer survival plots for cardiovascular mortality according to SM plasma concentrations in patients with stable (Figure 2a) and unstable angina (Figure 2b).
